# Plasticity after perceptual narrowing for voice perception: reinstating the ability to discriminate monkeys by their voices at 12 months of age

**DOI:** 10.3389/fpsyg.2013.00718

**Published:** 2013-10-09

**Authors:** Rayna H. Friendly, Drew Rendall, Laurel J. Trainor

**Affiliations:** ^1^Department of Psychology, Neuroscience and Behaviour, McMaster UniversityHamilton, ON, Canada; ^2^Department of Psychology, University of LethbridgeLethbridge, AB, Canada; ^3^Rotman Research Institute, Baycrest CentreToronto, ON, Canada

**Keywords:** voice discrimination, perceptual narrowing, infant development, plasticity, cross-species experience, learning

## Abstract

Differentiating individuals by their voice is an important social skill for infants to acquire. In a previous study, we demonstrated that the ability to discriminate individuals by voice follows a pattern of perceptual narrowing (Friendly et al., 2013). Specifically, we found that the ability to discriminate between two foreign-species (rhesus monkey) voices decreased significantly between 6 and 12 months of age. Also during this period, there was a trend for the ability to discriminate human voices to increase. Here we investigate the extent to which plasticity remains at 12 months, after perceptual narrowing has occurred. We found that 12-month-olds who received 2 weeks of monkey-voice training were significantly better at discriminating between rhesus monkey voices than untrained 12-month-olds. Furthermore, discrimination was reinstated to a level slightly better than that of untrained 6-month-olds, suggesting that voice-processing abilities remain considerably plastic at the end of the first year.

## INTRODUCTION

Human perception becomes specialized for socially relevant information in faces, voices, music, and language through a process of *perceptual narrowing, *whereby perception improves for native stimuli experienced in the environment, and becomes worse for foreign stimuli not experienced in the environment (for reviews, see Scott et al., 2007; Lewkowicz and Ghazanfar, 2009). Perceptual narrowing likely contributes to facilitating identification of individuals within one’s group and becoming a fully functioning member of that group. This specialization enables people to discriminate between individuals, identify group and species members, and discern who is from one’s own group and who is from outside one’s group in order to help inform decisions such as whether to approach or withdraw from a situation. A number of studies indicate that, although perceptual narrowing appears to be largely accomplished by the end of the first year after birth, a certain amount of plasticity remains beyond this age. Here we examine whether around 12 months of age a relatively small amount of experience with voices from a foreign species with which infants have little prior experience can reinstate the ability to discriminate pairs of voices from that foreign species.

An advantage for processing differences in native compared to foreign stimuli by 12 months of age has been documented across a number of domains, including faces (e.g., Pascalis et al., 2002; Lewkowicz and Ghazanfar, 2006; Kelly et al., 2007; Pons et al., 2009; Simpson et al., 2010), voices (Friendly et al., 2013), music (e.g., Lynch et al., 1990; Hannon and Trehub, 2005a, b; Trainor, 2005; Trehub and Hannon, 2006; Hannon and Trainor, 2007), language (e.g., Werker and Tees, 1984; Kuhl et al., 1992, 2006; Polka and Werker, 1994; Tsao et al., 2000; Kuhl, 2004, 2008; Palmer et al., 2012; for reviews see Werker and Tees, 2005; Curtin and Werker, 2007), and even action (Loucks and Sommerville, 2012). For example, 6-month-olds are equally good at discriminating two monkey faces as they are at discriminating two human faces, but 9-month-olds and adults are much better with human faces (Pascalis et al., 2002). Similarly, in the language domain, 6-month-old infants are equally good at discriminating consonant speech sounds from two native or two foreign phonemic categories whereas 10- to 12-month-olds are much better with native categories (e.g., Werker and Tees, 1984; Kuhl et al., 2006).

Although less studied than speech and faces, voices are important social stimuli for infants. People can be identified by the unique characteristics of their voices, which is especially useful when visual cues are poor. Voices also provide cues to the listener about the size, gender, and age of a talker (e.g., Smith and Patterson, 2005). Recently, Johnson et al. (2011) found that by 7 months of age, infants exhibit an own-language effect when discriminating voices, just as they exhibit an own-race effect when discriminating faces, with better discrimination of voices speaking their native language compared to a foreign language. Subsequently, Friendly et al. (2013) found evidence of perceptual narrowing for voices toward the end of the first year after birth. Specifically, between 6 and 12 months of age, infants became significantly worse at discriminating between two foreign-species (rhesus monkey) voices, but marginally better at discriminating between two native-species (human) voices.

The important role of experience in perceptual narrowing is indicated by research showing that sensitivity to foreign stimuli can in some cases be *maintained* with exposure to a foreign stimulus during the period when loss typically occurs (e.g., Burns et al., 2003; Pascalis et al., 2005; Scott and Monesson, 2009, 2010) or *reinstated* with exposure to a foreign stimulus after the period of loss (e.g., Kuhl et al., 2003; Hannon and Trehub, 2005b; Anzures et al., 2012). For example, Pascalis et al. (2005) found that after 2 weeks of daily exposure to monkey faces at 6 months of age, followed by 2.5 months of less frequent exposure, 9-month-olds maintained the ability to discriminate a novel set of monkey faces at a level comparable to that shown at 6 months of age. With respect to reinstatement, infants who received training at 8- to 12-months of age with other-race faces (Anzures et al., 2012), musical (Hannon and Trehub, 2005b), or linguistic (Kuhl et al., 2003) stimuli foreign to their environment, demonstrated successful processing of those stimuli, whereas their untrained counterparts did not. In the present paper, we investigate the generality of reinstatement by testing whether experience with rhesus monkey voices around 12 months of age can reverse the decrement in ability to discriminate such voices that has been documented in previous research (Friendly et al., 2013).

In designing our training protocol, we considered two features that appear to be important for exposure or training in infancy to result in learning. The first is individual-level encoding (Archambault et al., 1999; Waxman and Braun, 2005; Scott et al., 2006, 2008; Scott and Monesson, 2009, 2010). In the visual domain, Scott and Monesson (2009) repeated the Pascalis et al. (2005) monkey-face maintenance study, but used three different types of training regimen. One group of infants was trained at the individual level, similar to the infants in Pascalis et al.’s (2005) study, with parents labeling each monkey face with its own unique name (e.g., Dario, Flora, Boris, etc.). A second group of infants was trained at the category level, with parents labeling all monkey faces with the identifier “monkey.” The third group of infants was trained through passive viewing alone, with no label being read by parents during exposure. Interestingly, only the infants who received individual-level training with monkey faces showed maintenance of the ability to discriminate novel monkey faces at 9 months of age. Scott and Monesson (2009) concluded that individual-level exposure was critical for obtaining the effects found in Pascalis et al.’s (2005) study because it focused infants’ attention on what features were unique in each face, rather than on what the monkey faces had in common. Similarly, Anzures et al. (2012) successfully reinstated sensitivity to foreign-race faces in 8- to 10-month-olds after exposing them to daily videos of foreign-race women who introduced themselves by name. However, this was not compared to a no-name condition, making it unclear if labeling the faces by name influenced reinstatement in this study. Nevertheless, considering the findings from Scott and Monesson (2009), we designed our training procedure such that particular monkey voices would be associated with unique monkey names and characters.

The second feature that has been found to promote improved learning is social interaction. Kuhl et al. (2003) demonstrated that English-learning infants who were exposed to 12 sessions of Mandarin Chinese training between 9 and 10 months of age only showed a reinstatement in the ability to discriminate between Mandarin-specific phonemes at 10 to 11 months of age if they interacted with the Mandarin speakers in person. Infants who received the same training in the form of an audio-visual video or who received audio-alone training did not show a reverse in the decline of the ability to discriminate Mandarin phonemes. Similar benefits of social interaction have been found for native-language training using Baby Einstein^©^ videos, where infants required parental interaction in order to learn the words that were featured in the videos (Richert et al., 2010). Likewise, active musical interaction between infants and parents has been found to lead to earlier musical pitch enculturation compared to passive music listening (Gerry et al., 2012; Trainor et al., 2012). On the other hand, Anzures et al. (2012) found evidence of reinstatement, at 8 to 10 months of age, of sensitivity to foreign-race faces after 3 weeks of daily exposure to audio-visual videos of other-race females and Hannon and Trehub (2005b) found reinstatement of sensitivity to foreign musical rhythms in 12-month-old infants who passively listened to these rhythms at home on a CD. However, while parents were instructed to avoid drawing the infants’ attention to the music and instead to go about their regular routines, the CD may have been played during some type of social interaction between the parent and infant.

In the present study, we gave 11.5-month-old infants specific exposure to rhesus monkey voices under conditions that promoted individual-level encoding in the context of social interaction. In particular, we designed a storybook and accompanying audio CD narration that parents listened to with their infants twice a day for a 2-week period. The storybook contained a number of exemplars of each of four monkey characters’ voices. Following this exposure, we tested infants’ ability to discriminate a new set of monkey voices. We compared their discrimination of monkey voices to that of the 12- and 6-month-olds in Friendly et al. (2013) who did not receive any training. We aimed to determine whether this exposure would reinstate the ability to discriminate the monkey voices to the original level found at 6 months of age, before perceptual narrowing was fully underway.

## MATERIALS AND METHODS

### PARTICIPANTS

Twenty-four infants (mean age = 12.0 months, SD = 0.19 months at the time of testing; 10 females) received 2 weeks of monkey-voice training prior to testing (Trained-12 month Group). They were compared to two groups of infants from our previous study that were tested in the identical procedure, but who did not receive any training (Friendly et al., 2013). One group was also 12 months of age (*n* = 24, mean age = 12.0 months, SD = 0.19 months; 9 females; Untrained-12 month Group) and the other group was 6 months of age (*n* = 24; mean age = 6.1 months, SD = 0.22 months, 11 females; Untrained-6 month Group). Parents gave informed consent and reported normal hearing for all infants. Parents also reported all infants as hearing English 98–100% of the time in their home environment. An additional 20 infants across the three groups were excluded from the final sample due to fussiness (*n* = 4), failure to pass the familiarization phase of testing (see Procedure section 2.3.2 below; *n* = 7), receiving less than 23 training sessions (*n* = 5), being too old at time of testing (*n* = 1) and hearing non-English languages more than 2% of time in their home environment, as reported by parents (*n* = 3).

### STIMULI AND APPARATUS

#### Training stimuli

**FIGURE 1:**
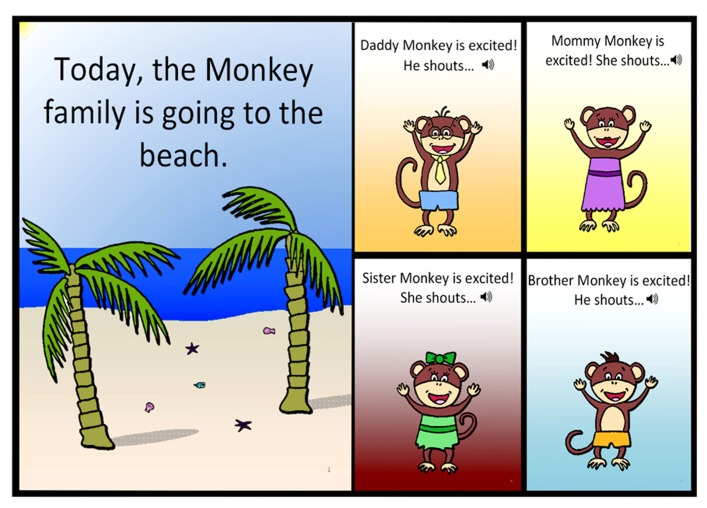
**An excerpt from the training stimulus storybook “Beach Day for the Monkey Family.”** A CD that contained the story narration and vocalizations belonging to four individual rhesus monkeys (six different coo tokens per monkey) accompanied the storybook. Each rhesus voice was always associated with the same monkey character in the story (either Daddy, Mommy, Sister or Brother Monkey), and the monkey characters were presented in the same order six times throughout the book. A sample of the storybook and CD can be found at:

A CD-narrated picture storybook was created for the monkey voice training. Entitled “Beach Day for the Monkey Family,” it contained colorful illustrations of four members of the Monkey Family going out for a day at the beach. Each monkey (labeled as Daddy, Mommy, Sister and Brother Monkey) was shown individually on a separate page, in consecutive order, six times throughout the storybook (for an example, see **Figure 1**). The CD-narration for the storybook was read by a monolingual English-speaking adult female and spoken in an infant-directed manner. Parents were instructed to listen to the accompanying CD and turn the page only at the sound of the chime, which occurred 4 s after the last vocalization on each page. The CD was designed so that every time one monkey was being viewed in the storybook, infants heard two vocalizations produced by a real rhesus macaque (*Macaca mulatta*). Thus, on the CD, each monkey character in the storybook was associated with the vocalizations of only one particular rhesus monkey. Twelve rhesus voice recordings were heard for each of the four rhesus monkey characters on the CD (6 tokens of the “coo” call category, heard two times each). The rhesus monkey recordings were obtained from author DR (for methodology on obtaining these recordings, see Rendall et al., 1996; Owren and Rendall, 2003), and edited using Cool Edit Pro [Syntrillium Software; sampling rate = 44.1 kHz, (intensity) resolution = 16-bit] and normalized for peak intensity across the sample. On the CD, the recordings of each monkey were ordered randomly, with the stipulation that the same “coo” token was never presented twice in a row and that the two tokens heard on each page formed a unique pair. The four rhesus monkeys on the CD were different from the monkeys used for testing. Those on the CD formed two matched pairs (Daddy/Mommy monkey; Sister/Brother monkey) such that the set of tokens for each voice in the pair were matched for mean duration (mean = 0.367 sessions, SD = 0.065 sessions; mean = 0.477 sessions, SD = 0.080 sessions, for the two pairs, respectively) and minimum (mean = 218.78 Hz, SD = 116.45 Hz; mean = 469.45 Hz, SD = 24.78 Hz), maximum (mean = 458.48 Hz, SD = 70.31 Hz; mean = 535.57 Hz, SD = 20.50 Hz) and mean (mean = 340.12 Hz, SD = 86.48 Hz; mean = 514.46 Hz, SD = 15.55 Hz) F0 (analyzed using Praat software‘s autocorrelation algorithm, F0 searched for between 100 and 600 Hz; Boersma and Weenink, 2009). An excerpt from the training storybook and CD (full version: 6 min 40 s in duration) can be found at: .

#### Testing stimuli

The rhesus monkey vocalizations used for testing were identical to those used in our previous study (see Friendly et al., 2013), but different from those used during training. Six vocal samples of the “coo” call from each of four female rhesus monkeys were obtained from author DR (see Rendall et al., 1996; Owren and Rendall, 2003), edited using Cool Edit Pro [sampling rate = 44.1 kHz, (intensity) resolution = 16-bit] and normalized for peak amplitude across the sample (Pair 1: voice 1 mean = 49 dB, range = 46–54 dB, voice 2 mean = 49 dB, range = 47–51 dB; Pair 2: voice 1 mean = 55 dB, range = 53–57 dB, voice 2 mean = 56 dB, range = 55–57 dB). Two pairs of primate voices (6 different “coo” call tokens for each individual monkey) were paired based on acoustic analyses using Praat software, such that their sets of tokens were matched for mean duration (mean = 0.30 sessions, SD = 0.047 sessions; mean = 0.27 sessions, SD = 0.061 sessions, for the two pairs, respectively) and minimum (mean = 282.36 Hz, SD = 66.28 Hz; mean = 502.48 Hz, SD = 18.93 Hz), maximum (mean = 351.84 Hz, SD = 49.87 Hz; mean = 562.59 Hz, SD = 32.49 Hz) and mean (mean = 320.50 Hz, SD = 48.72 Hz; mean = 542.04 Hz, SD = 26.30 Hz) F0. Four conditions (1A, 1B, 2A and 2B) were created for testing infants so that, for each voice pair (1 and 2), one voice in the pair served as the “change” voice and the other as the “background” voice for condition A (see Procedure). The change and background voices were switched for condition B.

### PROCEDURE

#### Training procedure

Two weeks prior to testing, infants in the Trained Group were mailed a package containing the illustrated storybook and accompanying CD. The package also contained a music and language questionnaire, daily reading log and instructions for the infant’s training schedule. The questionnaire asked what languages were spoken in the home and, for each, the proportion of time it was spoken, as well as whether infants attended music classes, whether parents played musical instruments, how often parents sang to their infants each week, and how often the infants listened to music each week. Parents were instructed to play the CD twice a day at home for 2 weeks, for a total of 28 training sessions, following along in the storybook with their infant. In order to ensure that the voice of each rhesus monkey on the CD was associated with a particular character in the book (labeled either Daddy, Mommy, Sister or Brother Monkey), parents were instructed to turn each page of the storybook only when they heard a musical chime sound. To make sure that infants were actively engaged during the monkey-voice training, parents were instructed to listen to the storybook together with their infant, interacting to engage their infant’s attention as much as possible. Infants were reported to have received between 23 and 29 sessions of training (mean = 28 sessions, approximately 6.5 min per session). One day after the completion of the 2-week training period, infants were brought into the lab for testing.

#### Testing procedure

Infants in the Trained Group were tested in the identical conditioned head turn (CHT) procedure as infants in the Untrained Groups from Friendly et al. (2013; also see Werker et al., 1998). Infants were assigned randomly to one of 4 stimulus conditions (1A, 1B, 2A or 2B), where the A and B conditions reversed which voice of the pair was the background and which the change voice.

During the testing phase of the CHT procedure, a loudspeaker located 90° to the infant’s left played the six *“coo”* tokens from the background voice repetitively in a quasi-random order such that the same token was never repeated consecutively (stimulus onset asynchrony = 1750 ms). The parent sat across from the experimenter with the infant seated on his/her lap and listened to masking music through headphones in order to eliminate potential parental influence on the infant’s behaviour. The experimenter likewise listened to masking music during testing. Throughout the experiment, tokens from the background voice were played continuously. The experimenter pressed one button when the infant was paying attention and facing forward (toward the experimenter), indicating to the computer that the infant was ready for a trial. There were 24 trials. Half (12) were control (no-change) trials that were indistinguishable from the repeating background. The other half (12) were change trials, on which the background voice was replaced by one of the six tokens of the changed voice for one repetition. Across the 12 trials, each of the six change-voice tokens was presented twice in a random order. The order of change and control trials was quasi-random, with the constraint that no more than two control trials were presented in a row. The experimenter pressed a second button when the infant made a head-turn response of 45 degrees or more to the left toward the speaker from which the sounds were played. Head turn responses occurring on control trials (i.e., false alarms) were not rewarded by the computer. In contrast, head turns on change trials (i.e., hits) that occurred within 1.5 s of the onset of the changed voice were rewarded by the computer with 2 s of an animated light and toy display. The proportion of hits and false alarms were converted into d-prime (d′) scores for data analysis.

Before infants began the testing phase of the CHT procedure, they first had to pass an initial training phase designed to familiarize them with the rule that when they made a head-turn response to a change from one monkey’s voice to another, they would be rewarded with an animated toy display. In this phase, only two of the six change-voice tokens were used and there were no control trials. Furthermore, during training the change voice was played, on average, 8 dB louder than the repeating background voice (see Stimuli and Apparatus) in order to make it a noticeable difference that would attract the infant’s attention to look toward the loudspeaker. In order to pass the familiarization phase and proceed to the testing phase, infants were required to make four correct head-turn responses in a row within 20 training trials. Infants who did not pass this training criterion were excluded from the final data set (see Participants). Once in the testing phase, all six “coo” tokens of the change were presented without the increase in intensity used during training.

## RESULTS

**FIGURE 2:**
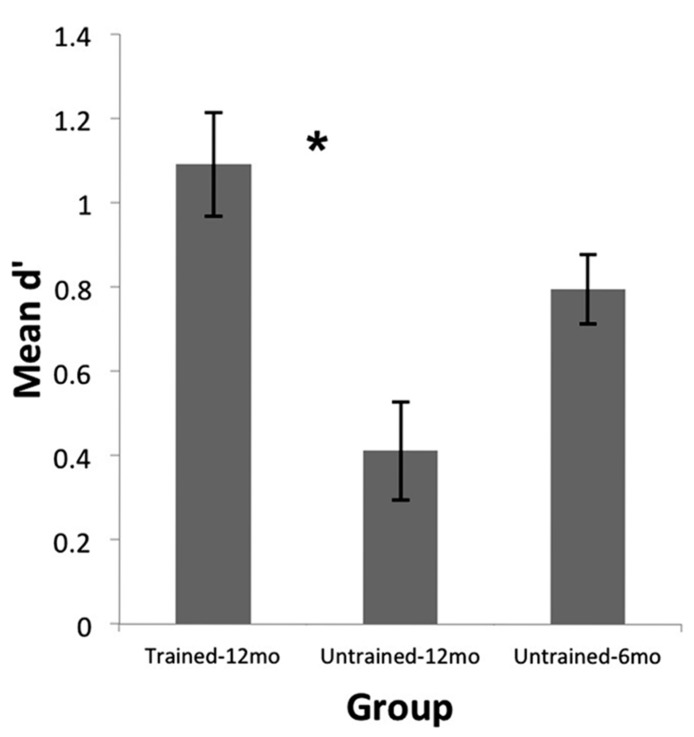
**Rhesus monkey voice discrimination abilities of 12-month-old infants (*n* = 24) who received monkey-voice training (Trained-12 month) in the present study compared to 6- (Untrained-6 month) and 12-month-old (Untrained-12 month) infants (*n* = 24 each) who did not receive training and were tested in a previous study (Friendly et al., 2013).** Discrimination was much better for trained than untrained 12-month-old infants (*p < 0.001). Trained 12-month-olds also preformed slightly better (*p* = 0.05) than untrained 6-month-old infants, suggesting that 2 weeks of exposure to monkey voices at 12 months reinstates processing of foreign-species voices to a level observed at 6 months, or better, before perceptual narrowing is fully underway. Error bars represent SEM.

Preliminary analyses revealed no significant differences in performance between male and female infants. As well, performance was not significantly related to whether or not infants attended music classes, whether parents played musical instruments, how often parents reported singing to their infants, and how often the infants were reported to listen to music. Thus these variables were not considered further in the following analyses. As can be seen in **Figure 2**, infants in the Trained-12 month group performed quite well at discriminating the monkey voices. Although all three groups performed significantly above chance levels (Untrained-12 month: *t*(23) = 3.53, *p* = 0.002; Untrained-6 month: *t*(23) = 9.70, *p* ’ 0.001; Trained-12 month: *t*(23) = 8.89, *p* ’ 0.001), a one-way ANOVA with group indicated a significant difference across groups in d′ scores, *F*(2,69) = 9.84, *p* ’ 0.001. A follow-up independent samples t-test indicated that infants in the Trained-12 month Group performed much better than age-matched infants in the Untrained-12 month Group, *t*(46) = 4.01, *p* ’ 0.001, Cohen’s *d *= 1.16. As well, a comparison of infants in the Trained-12 month Group to the younger infants who had not yet achieved perceptual narrowing (Untrained-6 month Group) indicated that the trained 12-month-olds actually performed slightly better than the untrained 6-month-olds, *t*(46) = 2.00, *p* = 0.05, Cohen’s *d *= 0.58. Furthermore, results from our previous research on the untrained infants showed that the ability to discriminate individual monkeys by voice decreased significantly between 6 and 12 months of age (Friendly et al., 2013). Together, these findings suggest that 2 weeks of exposure to monkey voices at 11.5–12 months of age (after perceptual narrowing has occurred) can reinstate sensitivity to voices from a foreign species to a level equivalent to or better than that observed at 6 months of age.

## DISCUSSION

In previous work, we showed that the development of specialization for own-species voice discrimination follows a pattern of perceptual narrowing, with a decrease in infants’ ability to individuate rhesus monkey voices between 6 and 12 months of age (Friendly et al., 2013). In the present paper, we found that after 2 weeks of twice-daily exposure to rhesus monkey voices in the form of a CD-narrated storybook, 12-month-old infants demonstrated significantly better discrimination of novel rhesus monkey voices not heard during training compared to 12-month-old infants who received no such training. Furthermore, the performance of the 12-month-olds who received exposure to the monkey voices was actually slightly better than that of untrained 6-month-old infants. The fact that monkey voice exposure enhanced discrimination of monkey voices in 12-month-olds to a level seen in 6-month-old infants indicates that the processes underlying perceptual narrowing for voice identification retain considerable plasticity at least until 12 months of age. This conclusion is consistent with studies in other domains that indicate that exposure to a socially relevant foreign stimulus can either maintain (Burns et al., 2003; Pascalis et al., 2005; Scott and Monesson, 2009, 2010) or reinstate (Kuhl et al., 2003; Hannon and Trehub, 2005b; Anzures et al., 2012) sensitivity past the period during which perceptual narrowing normally occurs.

Previous studies on face processing suggest that maintenance of sensitivity for individuating faces from a foreign species requires that the exposure to those faces be at an individual level, with different labels, such as names, being applied to the different faces in the exposure set (e.g., Scott et al., 2006; Scott and Monesson, 2009). These studies found that having infants simply observe foreign-species faces, or experience them in the context of a common label applied to all faces (e.g., “monkey”), does not lead to maintenance of the ability to discriminate these faces. As well, Anzures et al. (2012) found that exposure to labeled foreign-race faces at 8- to 10-months reinstates infants’ ability to recognize foreign-race faces, although they did not test under conditions with no labeling. Scott and Monesson (2009) suggest that labeling faces by name might draw infants’ attention to the differences between individuals, rather than to what the individuals have in common. On the other hand, phoneme categories in speech and metrical structures in music do not apply to individual people or individuals from other species (although changes in phonemes can signal changes in word meaning), and explicit individuation though the use of labels in these cases does not seem to be necessary for exposure to foreign categories or structures to disrupt perceptual narrowing (Kuhl et al., 2003; Hannon and Trehub, 2005b). The case of distinguishing individuals by their voice would seem to be similar to the case of distinguishing individuals by their face, suggesting that attention to differences between individuals during exposure might be critical for reinstating sensitivity to voices from a foreign species. This could be tested in future research with respect to distinguishing voices by using a training protocol that either applies no label at all or that applies the label “monkey” to each rhesus monkey voice sample during training.

Social interaction has also been identified as important for plasticity during the period of perceptual narrowing (Kuhl et al., 2003; Gerry et al., 2012). For example, Kuhl et al. (2003) found that interpersonal interaction between English-learning infants and Mandarin-speaking adults reinstated infant’s sensitivity to Mandarin phonemic distinctions after narrowing had occurred, whereas exposure to audio-visual and audio-alone recordings of these adults speaking Mandarin did not. On the other hand, Hannon and Trehub (2005b) found that 2 weeks of twice-daily passive exposure to foreign musical rhythms was sufficient to reinstate 12-month-olds’ ability to detect violations in foreign rhythmic structure. In the present study, infants receive training in the social context of parental interaction. It is possible that passive exposure to foreign stimuli can affect perceptual narrowing, but that exposure in a social context is more powerful. It remains for future research to determine whether the social interaction during the training phase of the present study was a necessary condition for the reinstatement of sensitivity to monkey voices. In order to investigate this, future studies could test training conditions in which parents are instructed to avoid interaction with their infant while experiencing the audiobook (audio-visual condition) or instructed to listen to the narration without looking at the storybook (audio-alone condition). If social interaction is as important for plasticity during perceptual narrowing for voices as it is for phonemic categories, then the infants in the audio-visual and audio-alone conditions should show no (or less) reinstatement of ability to discriminate monkey voices compared to the trained 12-month-olds in the present study.

In interpreting the effects of different kinds of experience on reinstatement of abilities at 12 month of age, it is important to consider the results from a study by Fair et al. (2012), in which sensitivity to distinctions in foreign-species (monkey) faces at 12 months was observed without a prescribed at-home training period, by simply by extending the length of the familiarization period at the time of testing. Specifically, Fair et al. (2012) demonstrated that 12-month-olds showed no evidence of discriminating unfamiliar monkey faces after 20 s of familiarization, but did discriminate them after 40 s of familiarization. This extended familiarization period could be considered a relatively brief form of training, but it is surprising that reinstatement could be achieved after such a brief training.

A final question concerns the age range over which the window of plasticity remains open with respect to learning to discriminate foreign voices. In other domains, there is evidence that some plasticity remains throughout the lifespan in that exposure to foreign stimuli in childhood (e.g., Feinman and Entwisle, 1976; Cheour et al., 2002; Shestakova et al., 2003; Wang and Kuhl, 2003; Sangrigoli et al., 2005; Macchi Cassia et al., 2009) or adulthood (e.g., Tees and Werker, 1984; Bradlow et al., 1999; McCandliss et al., 2002; Iverson et al., 2005; Pruitt et al., 2006; Scott et al., 2006, 2008; de Heering and Rossion, 2008; Kuefner et al., 2008; Zhang et al., 2009) results in improved processing of those stimuli, particularly if the training contains highly variable and numerous stimuli (Zhang et al., 2009), if differences between the stimuli are exaggerated (McCandliss et al., 2002), and if the person had some exposure to the stimuli earlier in life (e.g., Lenneberg, 1967; Tees and Werker, 1984; Newport et al., 2001; Sangrigoli et al., 2005; Macchi Cassia et al., 2009; Oh et al., 2010). However, completely native-like processing of foreign stimuli appears to be very difficult, if not impossible, to achieve in adulthood (e.g., Takagi and Mann, 1995; Flege et al., 1999; McCandliss et al., 2002; Takagi, 2002; Hannon and Trehub, 2005b; Iverson et al., 2005, for reviews see Birdsong, 2006; Hernandez and Li, 2007). Nevertheless, Sangrigoli et al. (2005) found native-like discrimination of Caucasian (French) faces by Korean adults who were adopted by French families between 3 and 9 years of age, suggesting that it is possible to demonstrate native levels of foreign-race face processing under certain conditions. In the present study, informal feedback from parents who participated with their infants suggests that parents had a difficult time distinguishing between the four monkey voices used in the storybook, even after listening to the story with their infant for the 2-week, twice-daily period. However, it is possible that with sufficient training adults could become proficient at discriminating foreign voices, as their ability to discriminate the monkey voices is poor, but above chance levels, without training. Again, this could be investigated in a future study.

In summary, perceptual narrowing achieved by the end of the first year after birth for discriminating voices can be modified by 2 weeks of exposure to voices from a foreign species, indicating a period of flexibility and plasticity following narrowing. It remains for future research to determine, (1) the time course of this plasticity across the lifespan, (2) effects of the social context, (3) whether individual-level training is important in perceptual narrowing for voice discrimination, (4) whether perceptual narrowing also occurs for other voice types (e.g., vocalizations from other species, sexes, races, and age groups), and (5) which particular acoustic characteristics of native and/or foreign voices, if any, promote the perceptual narrowing for voices observed in Friendly et al. (2013) and the reinstatement of foreign-voice discrimination in the present study.

## Conflict of Interest Statement

The authors declare that the research was conducted in the absence of any commercial or financial relationships that could be construed as a potential conflict of interest.
